# Extreme-QTL mapping of monepantel resistance in *Haemonchus contortus*

**DOI:** 10.1186/s13071-019-3663-9

**Published:** 2019-08-14

**Authors:** Simone Cristina Méo Niciura, Polyana Cristine Tizioto, Caroline Valério Moraes, Giovanna Gabrielle Cruvinel, Ana Cláudia Alexandre de Albuquerque, Raul Costa Mascarenhas Santana, Ana Carolina de Souza Chagas, Sergio Novita Esteves, Magda Vieira Benavides, Alessandro Francisco Talamini do Amarante

**Affiliations:** 1 Embrapa Pecuária Sudeste, Rodovia Washington Luiz, km 234, Fazenda Canchim, São Carlos, SP CEP 13560-970 Brazil; 2 NGS Soluções Genômicas, Rua Ajudante Albano, 847, Piracicaba, SP CEP 13416-030 Brazil; 3 Universidade Federal de São Carlos, Rodovia Washington Luiz, km 235, São Carlos, SP CEP 13566-905 Brazil; 4 Centro Universitário Central Paulista, Rua Miguel Petroni, 5111, São Carlos, SP CEP 13563-470 Brazil; 5 Faculdade de Medicina Veterinária e Zootecnia, Universidade Estadual Paulista (UNESP), Rua Prof. Doutor Walter Mauricio Correa, s/n, Botucatu, SP CEP 18618-681 Brazil; 6 Embrapa Pecuária Sul, Rodovia BR-153, Km 632,9, Vila Industrial, Bagé, RS CEP 96401-970 Brazil; 7 Instituto de Biociências de Botucatu, Universidade Estadual Paulista (UNESP), Rua Prof. Dr. Antônio Celso Wagner Zanin, 250, Distrito de Rubião Junior, Botucatu, SP CEP 18618-689 Brazil

**Keywords:** Anthelmintic resistance, Genome sequencing, Sheep gastrointestinal nematodes, Drug resistance, F2 mapping

## Abstract

**Background:**

*Haemonchus contortus*, a gastrointestinal nematode parasite of sheep, is mainly controlled by anthelmintics; the occurrence of anthelmintic resistance leads to treatment failures and increases economic burden. Because molecular mechanisms involved in drug resistance can be elucidated by genomic studies, an extreme quantitative trait locus (X-QTL) mapping approach was used to identify co-segregation of the resistance phenotype with genetic markers to detect the genome-wide variants associated with monepantel resistance in *H. contortus*.

**Methods:**

A cross between *H. contortus* isolates using parental susceptible (Par-S) males and monepantel resistant (Par-R) females resulted in SR progeny, while reciprocal cross resulted in RS progeny. Pools (*n* = 30,000) of infective larvae (L3) recovered from Par-R, and from SR and RS populations in the F3 generation, collected both before (unselected group) and 7 days after (selected group) selection with monepantel treatment in sheep hosts, were subjected to genome sequencing (Pool-Seq). Pairwise comparisons of allele frequencies between unselected and selected groups were performed for each population by Fisher’s exact test (FET) and for both populations combined by a Cochran-Mantel-Haenszel (CMH) test.

**Results:**

Mapping rates varied from 80.29 to 81.77% at a 90.4X mean coverage of aligned reads. After correction for multiple testing, significant (*P* < 0.05) changes in allele frequencies were detected by FET for 6 and 57 single nucleotide polymorphisms (SNPs) in the SR and RS populations, respectively, and by the CMH test for 124 SNPs in both populations. The significant variants located on chromosome 2 generated a selection signal in a genomic region harboring the mptl-1, deg-3 and des-2 genes, previously reported as candidates for monepantel resistance. In addition, three new variants were identified in the mptl-1 gene.

**Conclusions:**

This study expands knowledge on genome-wide molecular events underlying *H. contortus* resistance to monepantel. The identification of a genome region harboring major genes previously associated with monepantel resistance supports the results of the employed X-QTL approach. In addition, a deletion in exon 11 of the mptl-1 gene should be further investigated as the putative causal mutation leading to monepantel resistance.

**Electronic supplementary material:**

The online version of this article (10.1186/s13071-019-3663-9) contains supplementary material, which is available to authorized users.

## Background

Global demand for animal protein, mainly for beef and lamb meat, is expected to increase in the next decade [1]. Considering that sheep and goats are smaller and have higher resistance to adverse conditions than cattle, the production of small ruminants is considered to be a less risky investment and an important resource of food and income, especially for smallholders [2]. However, infection by gastrointestinal nematodes is the main limitation for raising small ruminants worldwide. Moreover, *Haemonchus contortus*, a hematophagous and highly prolific helminth, is the most prevalent and pathogenic parasite of sheep in tropical areas. Parasitism by gastrointestinal nematodes causes a large economic burden, which results from anemia, reduced rates of weight gain, lower carcass value and death [3]. Furthermore, the use of ineffective treatments, which are a consequence of anthelmintic resistance, also leads to economic losses [4].

Severe levels of anthelmintic resistance were reported [5] before the development of amino-acetonitrile derivatives (AADs). Therefore, the AAD monepantel was launched as an alternative to control worms in flocks with multidrug resistance [6]. However, resistance to monepantel has already been reported in goats and sheep on several continents [7–9]; therefore, there is an urgent need for a new approach to detect resistance and to develop improved therapeutics for helminth control.

Resistance is an evolutionary process in which resistant individuals survive drug treatments and transmit their genes to the next generation [10]. After a few generations, the frequency of resistant individuals increases in the population [11], which leads to anthelmintic failure. Resistance may be caused by molecular changes in the genes encoding drug targets, leading to drug-specific resistance, or in genes involved in drug permeability and detoxification, leading to multidrug resistance [12]. Each anthelmintic has a target, and monepantel acts against a nematode-specific subtype of nicotinic acetylcholine receptor (nAChR), disturbing ion flux and paralyzing the nematode [6]. To date, the molecular mechanisms associated with monepantel resistance in *H. contortus* have been reported for the target genes mptl-1, des-2 and deg-3 [6, 13, 14].

Previous studies have identified several genes that contribute to the resistance phenotype in an additive inheritance mode; thus, anthelmintic resistance has been suggested to be a complex quantitative trait [15]. However, for benzimidazole (see [16] for a review) and ivermectin [17], there is strong evidence for the involvement of only one gene or locus with major effects. Thus, it is of great interest to progress from the candidate gene approach to investigate if other genes contribute to the phenotype of resistance. Furthermore, it is necessary to elucidate if the large number of resistance-associated polymorphisms and genes detected, which have not been replicated in independent studies or in different species, is due to the use of parasites without a controlled population structure, where it is not possible to differentiate genes of resistance from those related to diversity [18]. Thus, large-scale technologies, combined with the availability of reference genomic sequences for *H. contortus* [19, 20], and the applicability of genetic crosses between parasite populations with extreme phenotypes [21], have created new opportunities to study genomic molecular mechanisms involved in anthelmintic resistance in this nematode species.

For this purpose, an experimental genetic cross, followed by genotypic and phenotypic characterization of progeny, is necessary to assess genome regions with the co-segregation of phenotypic traits with genetic markers [22]. Bulk segregant analysis (BSA) is a linkage mapping approach that compares differences in allele frequency measured by quantitative genotyping in populations of progeny with similar phenotypes in order to identify a quantitative trait locus (QTL) [22]. An extension of BSA, termed extreme-QTL (X-QTL), was developed in yeast to enlarge sample size, increasing the power to detect multiple loci with minor effects, and allowing complex traits to be mapped [23]. In parasites, X-QTL, also termed F2 mapping, has been used to detect oxamniquine resistance genes in *Schistosoma mansoni* [24] and ivermectin resistance genes in *Teladorsagia circumcincta* [25].

X-QTL involves three steps: (i) generation of segregating populations; (ii) selection by drug treatment, to enrich progeny pools for a particular phenotype, resulting in selected and unselected progeny with equal representation of allele frequencies across the genome, except for regions containing the genes that underlie the selected trait; and (iii) quantitative assessment of genome-wide allele frequencies in pooled samples by microarray-based genotyping or genome sequencing (Pool-Seq) [22–24]. In addition, Pool-Seq is an effective strategy to reduce costs in studies mapping the genotype-phenotype of variants [22].

In the present study, two *H. contortus* isolates with extreme phenotypes of susceptibility (S) and resistance (R) to monepantel were reciprocally crossed, resulting in two populations: one inheriting alleles of resistance from the mother (SR) and the other inheriting alleles of resistance from the father (RS). SR and RS progenies in the F3 generation were recovered as larvae (*n* = 30,000) before (unselected group) and after (selected group) monepantel treatment of sheep hosts and submitted to Pool-Seq. Thus, unselected and monepantel-selected progenies of *H. contortus* in the F3 generation were used as extreme phenotypes for the present X-QTL mapping. Then, differences in allele frequencies between unselected and selected worm pools for each population individually and for both combined populations were investigated to detect genome-wide events involved in the resistance of *H. contortus* to monepantel.

## Methods

### *Haemonchus contortus* isolates

Embrapa2010 and Botucatu are *H. contortus* isolates maintained and stored at Embrapa Pecuária Sudeste. Embrapa2010 is a monepantel-susceptible population isolated in Sao Carlos, SP, Brazil [26] before the release of monepantel in the Brazilian market. Botucatu is a field-selected monepantel resistant population isolated in Botucatu, SP, Brazil [27].

### Genetic cross

**Fig. 1:**
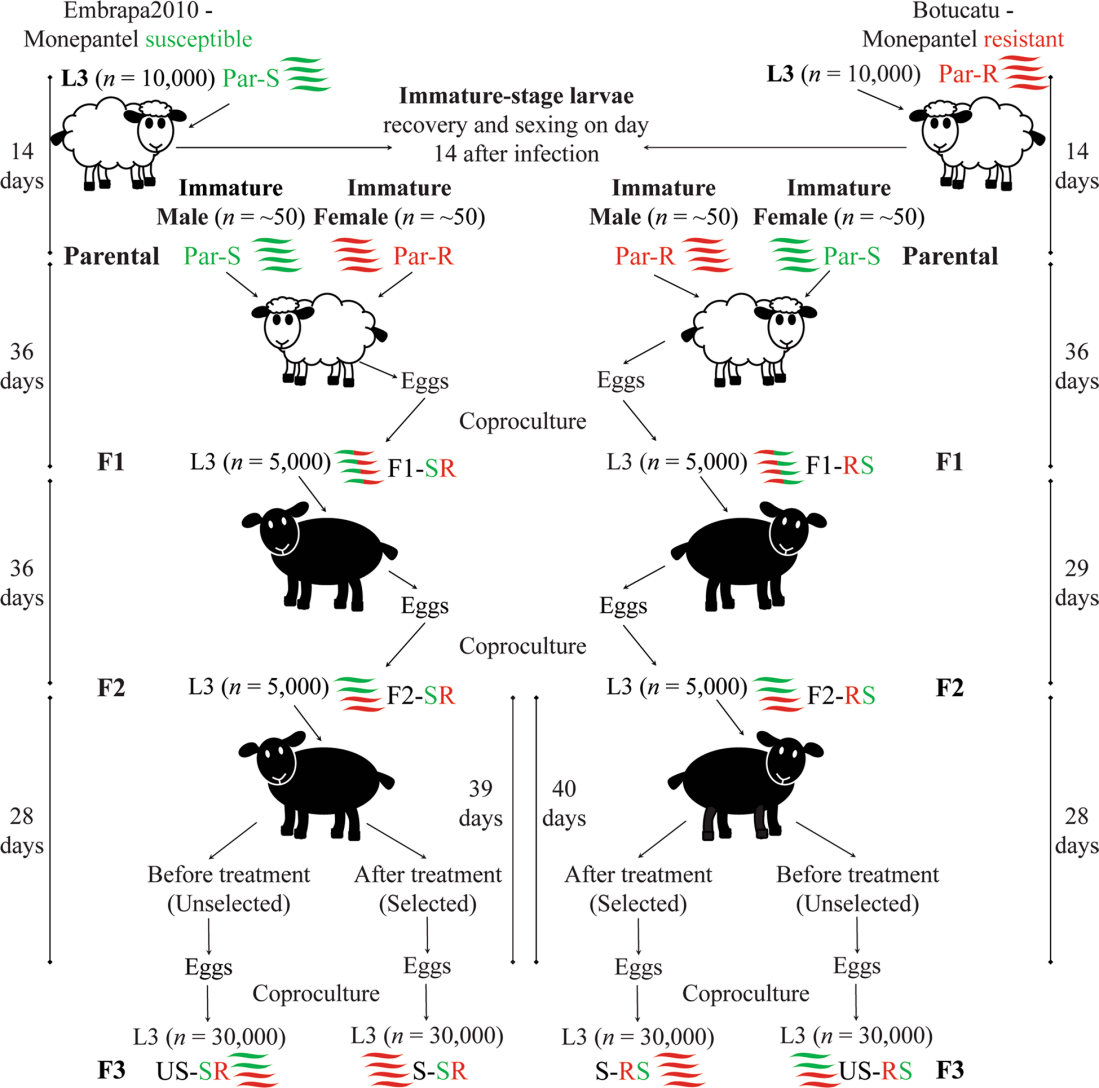
Genetic cross between *Haemonchus contortus* isolates with extreme phenotypes for monepantel resistance. Sheep hosts were infected with 10,000 infective larvae (L3) of *H. contortus* from monepantel-susceptible (Par-S L3, in green) or monepantel-resistant (Par-R L3, in red) isolates. Hosts were euthanized after 14 days of infection and immature-stage larvae collected from the abomasum were sexed. Approximately 50 male and female *H. contortus* immature-stage larvae of the susceptible isolate (Par-S immature) were surgically inoculated into the abomasum of another sheep host as the parent to be crossed, respectively, with ~ 50 females and males of the resistant isolate (Par-R immature), resulting in two F1 progenies (F1-SR and F1-RS) after 36 days. The F1 generation was recovered as eggs on feces and cultured to L3, and approximately 5000 L3 were used to orally infect new sheep hosts for F1 intercrossing. Then, the obtained descendants (F2 generation; after 29–36 days) were used (*n* = 5000) to orally infect new sheep hosts. Sheep hosting the *H. contortus* F2 generation were treated with 2.5 mg/kg monepantel. Before treatment, eggs recovered on feces (after 28 days) were cultured to L3 to obtain the unselected group (US-SR and US-RS), consisting of a mix of susceptible and resistant worms of the F3 generation progenies. The same process was performed after monepantel treatment, resulting in the selected group (S-SR and S-RS), consisting of only surviving resistant worms, recovered after 39–40 days. Pools of 30,000 *H. contortus* L3 from the parental resistant isolate (Par-R) and from unselected and selected F3 generation progenies (US-SR, US-RS, S-SR and S-RS) were subjected to DNA extraction and genomic sequencing (Pool-Seq). White sheep: Ile de France breed; black sheep: Santa Ines breed

The experimental design used for the genetic cross is described in Fig. 1. Briefly, males and females from Embrapa2010-susceptible and Botucatu-resistant *H. contortus* isolates were used as parents (Par-S and Par-R, respectively). Then, in sheep hosts, Par-S immature-stage larvae males were crossed with Par-R immature-stage larvae females, and Par-R immature-stage larvae males were reciprocally crossed with Par-S immature-stage larvae females to generate two F1 generation progenies (F1-SR and F1-RS, respectively), resulting in the inheritance of resistance alleles from maternal and paternal lineages, respectively. Subsequently, each F1 progeny was intercrossed to generate F2 progenies (F2-SR and F2-RS, respectively). Then, each F2 progeny in sheep hosts was subjected (selected group, S: S-SR and S-RS, respectively) or not (unselected group, US: US-SR and US-RS, respectively) to drug selection. Then, descendants from the F2 intercrossing or the F3 generations were recovered as eggs on feces, cultured to the third-stage infective larvae (L3), and pooled for DNA extraction and genomic sequencing (Pool-Seq).

Initially, housed Santa Ines or Ile de France sheep hosts, with no access to pastures, were treated with 10% trichlorphon (97 mg/kg; Neguvon®, Bayer, Belford Roxo, Brazil) to clear natural infection with gastrointestinal nematodes, as confirmed by two fecal examinations, 2-weeks apart. Based on previous studies [28], Ile de France, a sheep breed with low parasite resistance, presents higher fecal egg counts after artificial infection compared to Santa Ines sheep. Thus, Ile de France sheep were used as hosts during the most laborious experimental steps (involving euthanasia and surgery) to assure the recovery of a larger number of parasites. The following steps, involving oral infection and the collection of eggs from feces, were performed in Santa Ines sheep hosts.

For artificial infection, one sheep host was orally infected with 10,000 L3 of each *H. contortus* isolate (Par-S or Par-R) and euthanized after 14 days of infection for the collection of immature-stage larvae from the abomasum [21]. After immature-stage larvae were sexed, ~ 50 males from the Par-S isolate and ~ 50 females from the Par-R isolate, consisting of the parental generations, were surgically inoculated into the abomasum of a new sheep host. The reciprocal cross was performed in a different sheep host using Par-R males and Par-S females. After parental crossing, descendants or F1 progeny were recovered as eggs from feces and cultured to the L3 stage. Then, new sheep hosts were infected orally with 5000 L3 for F1 progeny intercrossing; the descendants resulted in F2 progeny, which were used to infect new sheep hosts.

### Drug selection

Each sheep hosting the F2 progeny was treated with monepantel (2.5 mg/kg; Zolvix®, Novartis, Basel, Switzerland). Eggs collected from feces and cultured to L3 from descendants of F2 progenies (the F3 generation) before monepantel treatment resulted in the unselected group, consisting of both susceptible and resistant L3 (US-SR and US-RS). Samples collected after sheep hosts were treated with monepantel resulted in the selected group, consisting of surviving resistant L3 only (S-SR and S-RS) (Fig. 1).

Regarding the parental, F1 and F2 *H. contortus* generations, a fecal egg count reduction test (FECRT) in sheep hosts [29] was used to estimate the reduced fecal egg count 7 days after monepantel treatment.

### DNA extraction and genomic sequencing

*Haemonchus contortus* L3 from the parental resistant (Par-R) isolate and from the unselected (US) and selected (S) progenies in the F3 generation (US-SR, US-RS, S-SR and S-RS) were collected in pools of 30,000 individuals and DNA was extracted using an organic solvent [30]. Briefly, L3 larvae pools were submitted to exsheathing with 0.125% sodium hypochlorite, washed by centrifugation (14,000×*g* for 5 min), frozen in liquid nitrogen and thawed five times, and incubated in digestion buffer (10 mM Tris-HCl, 10 mM EDTA, 50 mM NaCl, 2% SDS, 40 mM DTT and 0.4 mg/ml proteinase K) at 56 °C overnight. Then, DNA was extracted with phenol:chloroform:isoamyl alcohol (25:24:1), washed with 100% isopropanol and 70% ethanol, incubated in TE (10 mM Tris and 1 mM EDTA) with 10 µg/ml RNase at 37 °C for 1 h and stored at – 20 °C. The purity and integrity of DNA was evaluated by agarose gel electrophoresis, spectrophotometry (NanoDrop 2000, Thermo Fisher Scientific, Waltham, USA) and fluorimetry (Qubit 2.0, Thermo Fisher Scientific).

DNA was sheared by sonication into fragments of approximately 350 bp, subjected to library construction using a NEBNext DNA Library Prep Kit (New England Biolabs, Ipswich, USA), and verified by fluorimetry (Qubit, Thermo Fisher Scientific), micro-capillary electrophoresis (Agilent 2100 Bioanalyzer, Agilent, Santa Clara, USA) and qPCR. Paired-end PE 150 bp sequencing of pooled samples (Pool-Seq) was performed using the Illumina sequencing platform HiSeq X by Novogene Corporation (Beijing, China).

### Quality control, alignment and annotation

Novogene Corporation performed initial bioinformatic analyses as follows: Q-score calculation, determination of the distribution of the sequencing error rate, and data filtering using an in-house script. For data filtering, paired reads were discarded when either read was contaminated by adapter, when uncertain nucleotides (N) constituted more than 10% of either read, and when low-quality nucleotides (Q ≤ 5) constituted more than 50% of either read.

After quality control, effective sequencing data were aligned with the version 4 assembly of the MHco3(ISE).N1 [13] *H. contortus* reference sequence (BioProject PRJEB506; ftp://ftp.ebi.ac.uk/pub/databases/wormbase/parasite/releases/WBPS12/species/haemonchus_contortus/PRJEB506/haemonchus_contortus.PRJEB506.WBPS12.genomic.fa.gz) through BWA (parameters: mem -t 4 -k 32 -M, version 0.7.8-r455) [31]. Mapping rate and coverage were counted, and duplicates were removed using SAMtools (version 0.1.19-44428cd) [32] and merged using Picard (version 1.111; http://broadinstitute.github.io/picard/). Multi-sample variant calling was performed using SAMtools, and the ‘samtools mpileup’ command was used to generate the VCF format. Variants were filter by quality (Q ≥ 30), sequencing depth (DP ≥ 10), MAF ≥ 0.05 and call rate ≥ 0.95.

SnpEff (version 4.3T) [33] was used for annotation, utilizing the GFF3 file (ftp://ftp.ebi.ac.uk/pub/databases/wormbase/parasite/releases/WBPS13/species/haemonchus_contortus/PRJEB506/haemonchus_contortus.PRJEB506.WBPS13.annotations.gff3.gz).

### Genetic differentiation and allele frequency analyses

We expected to identify genome regions underlying monepantel resistance by detecting increased genetic diversity (F_ST_) or significant differences in the allele frequencies of variants across the genome by comparing unselected (US) with selected (S) pools in the F3 generation of SR and RS *H. contortus* populations. To achieve this, Pool-Seq was designed to obtain estimates from a large number of individuals (*n* = 30,000), and PoPoolation2 [34] was used to analyze genetic differentiation and per base changes in allelic frequencies to identify variants associated with monepantel resistance.

Initially, the mpileup file was converted into a synchronized file (popoolation2 mpileup2sync.jar; -min-qual 20). Then, PoPoolation2 was used to assess pairwise genetic differentiation between US and S groups throughout the genome for each SR and RS population by calculating the fixation index (F_ST_) (popoolation2 fst-sliding.pl; -pool-size 30,000 -min-count 6 -min-coverage 20 -max-coverage 200), considering window sizes of 1 bp, 500 bp, 1 kb and 10 kb and step sizes of 1, 100, 1000 and 5000.

Similarly, the statistical significance of differences in allele frequencies between US and S groups for each SR and RS population was determined using Fisher’s exact test (FET) (popoolation2 fisher-test.pl; -min-count 6 -min-coverage 30 -max-coverage 200 -window-size 1 -step-size 1 -suppress-noninformative). Then, aiming to detect consistent changes in allele frequencies common to both SR and RS populations, the Cochran–Mantel–Haenszel (CMH) test [35] (popoolation2 cmh-test.pl; -min-count 12 -min-coverage 35 -max-coverage 200 -population 1-2,3-4) was used for the pairwise comparison [36] between the US and S groups. All *P*-values obtained with FET and the CMH test were corrected for multiple testing (FDR < 0.05) [37]. Finally, single nucleotide polymorphisms (SNPs) with significant differences in allele frequencies were mapped back to the resistant parental (Par-R) genome to confirm that the resistant-associated allele was derived from this parent.

The description of genes harboring significant SNPs was obtained in WormBase ParaSite, version 13 (https://parasite.wormbase.org/index.html), and orthologous genes in *Caenorhabditis elegans* were obtained in WormBase (https://wormbase.org).

## Results

### Generation and validation of the genetic cross

The X-QTL procedure (Fig. 1), performed to generate samples for sequencing, lasted between 119 to 125 days, from the first infection of sheep hosts with Par-S and Par-R L3 *H. contortus* parental isolates to the recovery of eggs in feces from the selected groups (S-SR and S-RS) in the F3 generation.

Monepantel efficacy in *H. contortus*, assessed by FECRT, was 100% for Par-S and 0% for Par-R in the parental generation, 55% for F1-SR and 72% for F1-RS in the F1 generation, and 0% for F2-SR and 29% for F2-RS in the F2 generation.

### Genomic sequencing

Sequencing quality was confirmed by a Phred score of Q30 to Q40 and an expected distribution pattern of error rates. Raw data filtering (185.3 G) resulted in an average of 99.5% clean data (184.4 G or 99.7X coverage), which indicated the production of sufficient data.

Mapping rates to the *H. contortus* reference sequence varied from 80.29 to 81.77%, with a mean coverage of aligned reads at 90.4X. Similar amounts of raw and clean data, and average depths, were obtained for all sequenced samples. After mapping, a total of 18,796,507 passing filter variants were detected, which included 1,348,383 variants on chromosome X, and from 3,264,078 variants on chromosome 2 to 3,747,948 on chromosome 4. These variants consisted of 18,425,482 SNPs, 197,183 insertions and 173,842 deletions. With an effective genome length of 283,439,308 bp, one variant was detected every 15 bases, and the alternative allele was fixed in 185,310 to 350,789 variants. Most variants were annotated by predicted impact effect as modifiers (95.7%), mainly located in the intergenic (27.1%), intron (26.7%), upstream (20.7%) and downstream (20.4%) regions, and by functional class as silent (74.9%).

### X-QTL analysis

Pairwise comparisons between unselected (US) and selected (S) worm pools in each population were used to assess genetic diversity (F_ST_) and to detect differences in allele frequencies per base (FET) using PoPoolation2. F_ST_ values for each SR and RS population ranged from 0.06 to 0.37 using different window and step sizes; however, F_ST_ > 0.3 was only detected for SNPs analyzed individually (window size of 1 bp and step size of 1).

Regarding differences in allele frequencies assessed by FET, after correction of *P*-values for multiple testing (*P* < 0.05), six variants were detected in the SR population (Additional file 1: Table S1) and 57 variants were detected in the RS population (Additional file 2: Table S2). Differences in allele frequency after *P*-value correction for multiple testing common to both populations (SR and RS) using the CMH test were detected for 124 significant (*P* < 0.05) variants (Additional file 3: Table S3).

**Fig. 2:**
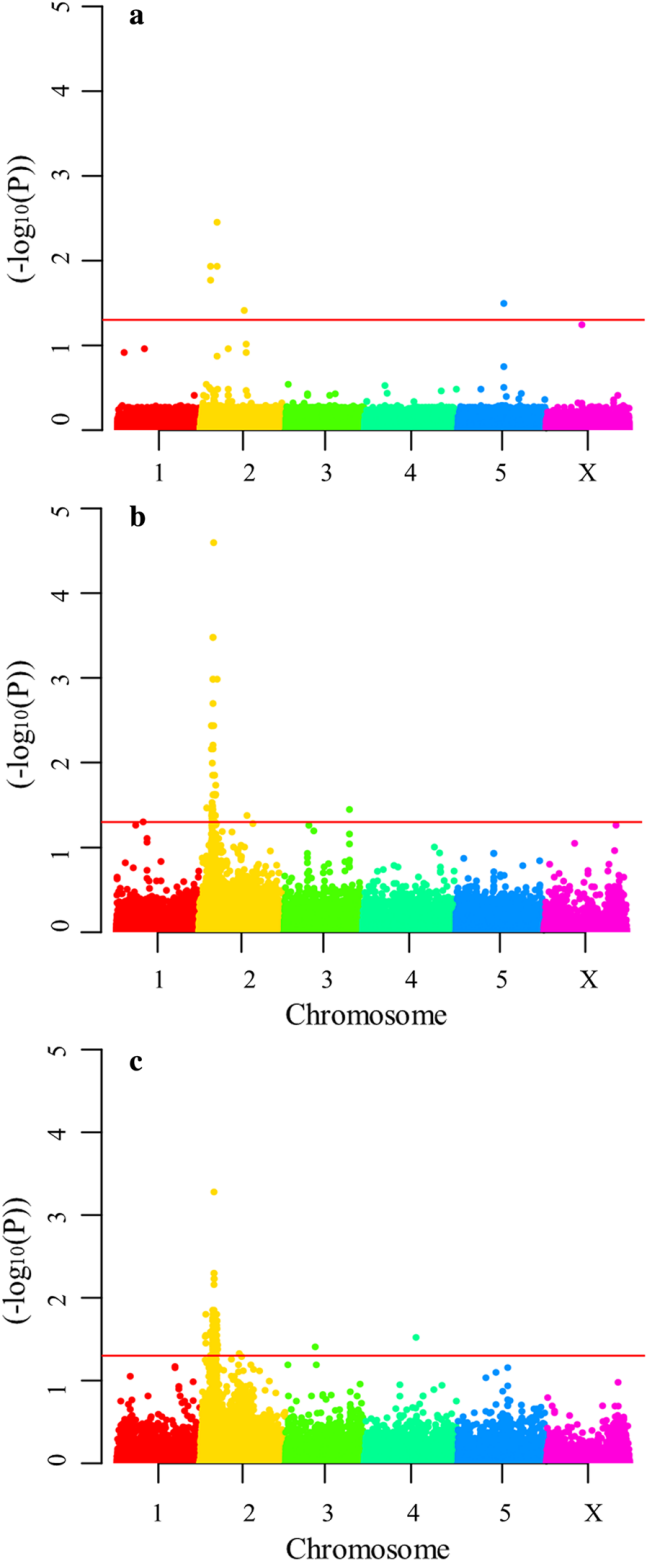
Extreme quantitative trait locus (X-QTL) mapping of monepantel resistance in *H. contortus*. Adjusted *P*-values for differences in allele frequencies between unselected and monepantel-selected worm pools plotted across the five autosomes and X chromosome of *H. contortus* for SR (**a**), for RS (**b**) and for both SR and RS (**c**) populations. The red line corresponds to correction for the false discovery rate (FDR). *Abbreviations*: SR, progeny in the F3 generation after crossing susceptible parental males with resistant parental females; RS, progeny in the F3 generation after crossing resistant parental males with susceptible parental females

Differences in allele frequencies (-log_10_ adjusted *P*-value) between unselected and selected pools for each variant were plotted across *H. contortus* chromosomes (Fig. 2), providing evidence for a selection signal on chromosome 2. When each population was analyzed individually, a weak signal and few significant variants were detected for the SR population (Fig. 2a): two variants 5 bp apart (at positions 5,746,092 and 5,746,097), two variants 32 bp apart (at positions 9,341,108 and 9,341,140) and one variant at position 24,373,119 on chromosome 2; and one variant at position 25,384,592 on chromosome 5. For the RS population (Fig. 2b), a strong peak consisting of 53 significant variants was detected on chromosome 2, in a region of 2.4 Mb (from position 7,013,333 to 9,403,437). In addition, isolated variants were detected on chromosome 2 at positions 4,403,522, 10,199,955 and 26,770,651, and on chromosome 3 at position 36,273,321. Considering both populations (Fig. 2c), a significant enrichment of alleles from the resistant parent was detected on chromosome 2, as shown by a *P*-value peak for 122 variants. Furthermore, one significant variant was detected on chromosome 3 at position 1,647,363 and another variant on chromosome 4 at position 2,863,865 (Fig. 2c).

Subsequently, SnpEff impact prediction was used to investigate the potential effect of all significant variants detected in SR (Additional file 1: Table S1), in RS (Additional file 2: Table S2) and in both SR and RS (Additional file 3: Table S3) populations. Most variants presented a predicted modifier effect (upstream, downstream, intron and intergenic) or were of low impact (synonymous, and splice region and intron variants). Only one variant, detected by the CMH test, was predicted to have a moderate impact: a missense SNP located on chromosome 3 at position 1,647,363 in the non-annotated HCON_00077020 gene (Additional file 3: Table S3). In addition, most of the detected variants were located in non-annotated genes or in genes with no orthologs in *C. elegans* (Additional file 1: Table S1, Additional file 2: Table S2 and Additional file 3: Table S3).

Based on the results from the CMH test (Fig. 2c), when all 122 significant variants detected on chromosome 2 were considered, the peak corresponded to a region of 6.8 Mb (from position 3,074,289 to 9,885,892). However, when only 100 kb windows containing at least one significant variant were considered, a 1.5 Mb region comprising 98 significant variants (from position 7,201,718 to 8,727,463) was found. This region harbors 159 genes, including the mptl-1 (HCON_00039360), deg-3 (HCON_00039370) and des-2 (HCON_00039380) genes, which were previously reported as candidate genes for monepantel resistance.

The contribution of these candidate genes to the results obtained in the present X-QTL analysis was further investigated. First, we observed that these three genes are located on chromosome 2 in a region spanning 58.6 kb (mptl-1 from 7,740,175 to 7,756,500, deg-3 from 7,771,467 to 7,786,447 and des-2 from 7,786,541 to 7,798,762), which was enclosed in the region containing the selection signal for monepantel resistance on chromosome 2 (Fig. 2). Secondly, the variant with the most significant *P*-value by the CMH test was located at position 7,901,587, which is close (145.1 to 102.8 kb) to the end of the mptl-1, deg-3 and des-2 genes. In addition, a significant variant (at position 7,756,689) was detected upstream to the mptl-1 gene and intergenic to the mptl-1 and deg-3 genes (Additional file 3: Table S3).

To investigate the occurrence of new polymorphisms in the mptl-1, deg-3 and des-2 genes leading to disruptive effects in the encoded proteins, the impact prediction by SnpEff was assessed for all variants detected on these genes, including the non-significant variants. No variants with a predicted high impact effect were detected in the *H. contortus* population of the present study. However, three variants with moderate impact on protein effectiveness were detected for the mptl-1 gene in exon 11: a missense SNP (c.896C>G|p.Pro299Arg) at position 7,744,383; a 6 bp in-frame deletion (c.854_859delTGTCAA|p.Ser286_Met287del) at position 7,744,415; and a 6 bp in-frame deletion (c.852_857delATTGTC|p.Leu285_Ser286del) at position 7,744,420. These two deletions are located in a sequence in exon 11 encoding transmembrane domain 2 of the mptl-1 protein. In addition, a consistent change in frequency associated with monepantel resistance was observed in 852_857del: from 38.5 and 35.8% in unselected groups (US_SR and US_RS, respectively), to 64.7 and 53.2% in selected groups (S_SR and S_RS, respectively) and to 71.4% in the parental resistant isolate (Par_R).

## Discussion

To obtain a controlled crossed *H. contortus* population for genome-wide mapping of monepantel resistance, a resistant isolate was reciprocally crossed with a susceptible isolate, resulting in two populations: SR and RS. Parental resistant, and unselected and monepantel-selected SR and RS populations in the F3 generation were submitted to Pool-seq and X-QTL analyses. Pairwise comparisons between selected and unselected groups revealed changes in allele frequencies associated with resistance for several variants, leading to a selection signal on chromosome 2, in a genomic region containing the mptl-1, deg-3 and des-2 genes.

Controlled genetic crosses lead to a population structure that allows the differentiation of background genetic variation, which occurs in genetically distinct populations, from variations associated with drug resistance [17]. Several crossing strategies can be employed, and for the X-QTL mapping approach used here, an initial genetic cross of parental isolates with extreme phenotypes was followed by two cycles of intercrossing in the F1 and F2 generations. This last generation was submitted to one round of drug selection, yielding the F3 generation used in genome sequencing. Another strategy is introgression mapping, which has previously been used to analyze multidrug resistance in *T. circumcincta* [25] and ivermectin resistance in *H. contortus* [17]. For introgression mapping, the initial cross of parents with extreme phenotypes is followed by several backcrossing cycles with the susceptible parental, followed by several rounds of drug selection. Compared with introgression mapping, the X-QTL method is faster, requires a lower number of crossing cycles and uses parasites at earlier stages (L3 instead of immature-stage larvae collected from the abomasum) in most of the steps. In addition, for X-QTL, only a single euthanization and surgical procedure step in sheep hosts was necessary to recover immature-stage larvae for the initial genetic cross, whereas for introgression mapping, immature-stage larvae must be recovered before each backcrossing cycle. In addition, the use of samples in the L3 stage and from the same F3 generation progeny (recovered from the same sheep host before and after selection by monepantel treatment) prevented the euthanasia of four sheep hosts to collect adults from the abomasum in the unselected and selected groups. Consequently, the X-QTL approach minimized the number of sheep hosts, interventions (euthanasia and surgeries) in hosts and nematode crossing cycles, also reducing the time required to obtain samples for sequencing. However, a disadvantage of the employed X-QTL approach was that crossing the parasites for fewer cycles, comparing unselected (mix of susceptible and resistant) with selected (only resistant) worm pools instead of resistant with only susceptible parasites, and using a single round of drug selection resulted in a lower genetic diversity (F_ST_) and smaller differences in allele frequencies in pairwise comparisons. This weaker evidence of selection for F2 mapping compared to introgression mapping has previously been reported for ivermectin resistance in *T. circumcincta* [25].

The genetic cross for X-QTL mapping was validated by FECRT and monepantel efficacy rates were assessed across generations. Efficacy decreased from the susceptible parental isolate to the F1 and F2 generations. Monepantel efficacy was 0% for the parental resistant isolate, whereas it was 100% for the parental susceptible isolate. In the F1 generation, efficacy was 55% for the SR population and 72% for the RS population. The presence of resistant individuals in the F1 generation indicates that monepantel resistance is dominant in *H. contortus*. Monepantel resistance has been suggested to be recessive and polygenic, involving at least two loci, based on *in vitro* tests [38]. However, a posterior molecular study [14] using the same *H. contortus* population from the previous study [38] detected the presence of 50% wild type (or susceptible) alleles in the resistant populations suggesting that monepantel resistance is dominant. Another observation regarding the FECRT results in the present study was that the rates of monepantel efficacy in offspring from resistant females (SR) both in the F1 (55%) and F2 (0%) generations were lower than those in offspring from resistant males (RS) in the F1 (72%) and F2 (29%) generations. These differences in the FECRT between SR and RS in the F1 and F2 generations indicate that the inheritance of resistance alleles from the maternal lineage resulted in higher monepantel resistance than inheritance from the paternal lineage. A maternal effect on thiabendazole resistance was previously reported in *H. contortus* [39]. However, considering the limited number of sheep hosts and FECRT trials employed herein, further studies are needed to confirm the inheritance mode of monepantel resistance in *H. contortus* regarding dominance and maternal effect.

After the sequencing data were aligned to the reference *H. contortus* genome, a large number of variants (one variant was detected every 15 bases) were detected. The samples used here were field-derived populations of *H. contortus* from Sao Paulo state, Brazil, while the reference genome was derived from the MHco3(ISE) isolate collected in East Africa and submitted to experimental passages and multiple generations of inbreeding in the UK [40]. Thus, the detection of a large number of variants may be due to the genetic diversity observed between populations of different geographical origins and also to differences in inbreeding status [17].

Genome regions associated with monepantel resistance were characterized by genetic differentiation, using single nucleotide and sliding-window F_ST_ approaches, and by differences in allele frequencies by FET and CMH tests. Considering that the extreme phenotypes in the present study consisted of unselected (a mix of resistant and susceptible worms) and monepantel-selected (only resistant worms) groups, and a dominant effect of monepantel resistance was observed, by which allele frequencies of at least 50% lead to the phenotype of resistance, F_ST_ approaches were not sensitive enough to differentiate the genomic regions under selection. Thus, differences in allele frequencies were estimated by FET for each population individually and by the CMH test for both populations combined. These analyses revealed a selection signal on chromosome 2, supporting evidence that a single major locus is the responsible for the establishment of monepantel resistance in *H. contortus*. This pattern was previously reported for other anthelmintic classes; including the association of benzimidazole resistance with the isotype-1 β-tubulin locus (see [16] for a review), and for ivermectin resistance with a locus on chromosome 5 in *H. contortus* [17].

Based on evidence that resistance can be caused by mutations on target-specific genes, leading to drug-specific resistance, and by variants on non-target-specific genes, leading to multidrug resistance [12], we investigated all significant variants detected by genome-wide X-QTL mapping. Notably, most variants occurred in non-annotated genes or in genes with no orthologs in *C. elegans*. In addition, none of the variants was predicted by SnpEff to have a disruptive high impact on proteins, and only one variant, located on chromosome 3, presented a predicted moderate effect. These findings suggest that the detected variants may not be essential for parasites and may not be related to the mechanisms of resistance [41]. Thus, they might be molecular markers of monepantel resistance rather than causal mutations. Considering the X-QTL approach employed here, in which unselected individuals (consisting of a mix of susceptible and resistant parasites) were compared to monepantel-selected individuals (consisting of only resistant parasites), and also because *P*-values were corrected for multiple testing, only very large differences in allele frequencies could be detected. Assuming this, we suggest that the detected variants are hitchhiking with a causal mutation that leads to monepantel resistance, creating a selection signal. In addition, as larger differences in allele frequencies are presented between groups, there might be tendency towards fixation in the resistant population. During a hard sweep, a new mutation conferring selective advantage arises and is driven to fixation, while adjacent genetic polymorphisms hitchhike together with the beneficial allele and both increase in frequency [42]. Thus, these findings suggest the occurrence of a hard selective sweep associated with monepantel resistance in *H. contortus*, which should be further investigated by appropriate analyses of genetic diversity, linkage disequilibrium or frequency spectrum [42, 43].

Considering that the monepantel target protein is a nematode-specific subtype of the nicotinic acetylcholine receptor (nAChR), and that mutations and altered expression of the mptl-1, deg-3 and des-2 genes, members of the DEG-3 subfamily of nAChR, were associated with *H. contortus* resistance to monepantel [6, 13, 14], the selection signal detected on chromosome 2 was further explored. Using data from the CMH test, the peak of significant *P*-values comprised a 1.5 Mb region on chromosome 2 in which the three major candidate genes (mptl-1, deg-3 and des-2) for monepantel resistance are located. The identification of a genomic region containing the putative candidate genes provides great confidence in our X-QTL approach, and further supports that alleles in one or more of these genes are responsible for monepantel resistance.

If differences in allele frequencies between unselected and selected groups were not high enough, potential causal mutations may not have been detected as significant by the X-QTL approach; therefore, we searched for all disruptive mutations present in the three candidate genes. No mutations predicted to have a high impact were detected; however, three mutations (a missense SNP and two deletions in the region encoding for the transmembrane 2 domain) in exon 11 of the mptl-1 gene, leading to a moderate impact on protein effectiveness, were detected in our *H. contortus* population. In previous studies, three missense mutations in the transmembrane 2 domain were detected in AAD-resistant *C. elegans* [6]; however, no variants in exon 11 of the mptl-1 gene have previously been reported in *H. contortus* [13, 14]. Furthermore, loss of amplification of a segment spanning intron 11 and exon 11 of the mptl-1 gene has been described [6] using PCR primers designed to a location upstream to the site of the variants described in the present study. Thus, the three variants detected in exon 11 of mptl-1 are reported for the first time in *H. contortus*.

Furthermore, to infer whether these three new mutations may lead to monepantel resistance in our population, their frequencies were assessed in unselected and selected groups and in the parental resistant isolate. The deletion at position 7,744,420 (852_857del), occurring in a region encoding an important domain (transmembrane domain 2) of the mptl-1 protein, presented consistent changes in allele frequencies between unselected and selected groups and in frequencies exceeding 50% in resistant (S-SR, S-RS, and Par-R) populations, consistent with a dominant effect of monepantel resistance, as suggested by the FECRT results. Transmembrane domain 2 of the nAChR folds into an α-helix [44] and plays a critical role during acetylcholine receptor-ligand gating [45], donating residues that line the ion channel [46]. Thus, we considered this deletion to be a putative causal mutation leading to monepantel resistance in our population and also driving the selection signal on chromosome 2. However, as this is an in-frame deletion that does not lead to protein truncation, further *in silico* and functional analyses should be performed to investigate the impact on the mptl-1 secondary and tertiary structures and protein function and the association with resistance.

## Conclusions

The X-QTL mapping approach detected a selection signal on chromosome 2, which is characterized by a potential hard selective sweep, indicating that a single major locus on chromosome 2 is involved in monepantel resistance in *H. contortus*. In addition, a deletion in a region of exon 11 encoding transmembrane domain 2 of the mptl-1 protein was suggested as a putative dominant mutation leading to monepantel resistance in our population. In conclusion, the results provided by this genome-wide study expand knowledge on the molecular basis of *H. contortus* resistance to monepantel.

## Additional files

**Additional file 1: Table S1.** Significant variants detected by X-QTL mapping of monepantel resistance in *Haemonchus contortus* from the SR population.

**Additional file 2: Table S2.** Significant variants detected by X-QTL mapping of monepantel resistance in *Haemonchus contortus* from the RS population.

**Additional file 3: Table S3.** Significant variants detected by X-QTL mapping of monepantel resistance in *Haemonchus contortus* from both the SR and RS populations.

**Additional file 4: Table S4.** Sample sequencing data available at the European Nucleotide Archive repository (study accession PRJEB33301).

## Data Availability

The datasets generated during the present study are available in the European Nucleotide Archive repository (http://www.ebi.ac.uk/ena/), under the study accession number PRJEB33301 (see Additional file 4: Table S4).

## Abbreviations

AADs: amino-acetonitrile derivatives
BSA: bulk segregant analysis
CMH: Cochran–Mantel–Haenszel
FDR: false discovery rate
FECRT: fecal egg count reduction test
FET: Fisher’s exact test
F_ST_: fixation index
F1-RS: F1 generation progeny generated after crossing male Par-R with female Par-S
F1-SR: F1 generation progeny generated after crossing male Par-S with female Par-R
F2-RS: F2 generation progeny from F1-RS intercrossing
F2-SR: F2 generation progeny from F1-SR intercrossing
L3: infective stage larvae
nAChR: nicotinic acetylcholine receptor
Par-R: parental generation of *H. contortus* resistant to monepantel
Par-S: parental generation of *H. contortus* susceptible to monepantel
Pool-Seq: genome-wide sequencing of pooled samples
QTL: quantitative trait locus
RS: progeny generated after crossing male Par-R with female Par-S
SNPs: single nucleotide polymorphisms
SR: progeny generated after crossing male Par-S with female Par-R
S-RS: selected F3 generation progeny from F2-RS intercrossing recovered 7 days after monepantel treatment of sheep hosts, consisting of resistant L3
S-SR: selected F3 generation progeny from F2-SR intercrossing recovered 7 days after monepantel treatment of sheep hosts, consisting of resistant L3
US-RS: unselected F3 generation progeny from F2-RS intercrossing recovered before monepantel treatment of sheep hosts, consisting of a mix of susceptible and resistant L3
US-SR: unselected F3 generation progeny from F2-SR intercrossing recovered before monepantel treatment of sheep hosts, consisting of a mix of susceptible and resistant L3
X-QTL: extreme quantitative trait locus
