# Supplementary material for: Extreme-QTL mapping of monepantel resistance in Haemonchus contortus
Source: Parasit Vectors. 2019 Aug 14;12:403. doi: 10.1186/s13071-019-3663-9 (PMC6693152; doi:10.1186/s13071-019-3663-9)
Supplement: Supplementary file 1 — Additional file 1: Table S1. Significant variants detected by X-QTL mapping of monepantel resistance in Haemonchus contortus from the SR population. [file 13071_2019_3663_MOESM1_ESM.docx]

**Additional file 1: Table S1** Significant variants detected by X-QTL mapping of monepantel resistance in *Haemonchus contortus* from the SR population

| **Ch** | **Pos** | **Ref** | **Alt** | **Res** | **US-SR** | **S-SR** | **Par-R** | ***P*adj** | **Ann** | **Gene** | **Name** | ***C. elegans***  **orthologous** |
| --- | --- | --- | --- | --- | --- | --- | --- | --- | --- | --- | --- | --- |
| 2 | 5746092 | G | T | T | 0:15:0:100 | 0:60:0:65 | 0:35:0:67 | 0.01699 | Upstream | HCON_00037550 | n/a | - |
| 2 | 5746097 | C | T | T | 0:14:99:0 | 0:59:63:0 | 0:34:67:0 | 0.01166 | Upstream | HCON_00037560 | n/a | - |
|  |  |  |  |  |  |  |  |  | Intergenic | HCON_00037550-HCON_00037560 | | |
| 2 | 9341108 | T | A | T | 84:92:0:0 | 22:118:0:0 | 227:138:0:0 | 0.01166 | Upstream | HCON_00040680 | n/a | - |
| 2 | 9341140 | T | C,A | T | 28:67:78:0 | 35:100:19:0 | 19:135:212:0 | 0.00352 | Downstream | HCON_00040660 | n/a | - |
|  |  |  |  |  |  |  |  |  | Downstream | HCON_00040670 | n/a | - |
|  |  |  |  |  |  |  |  |  | Intergenic | HCON_00040660-HCON_00040670 | | |
| 2 | 24373119 | A | G | G | 106:0:0:0 | 80:0:0:24 | 107:0:0:7 | 0.03870 | Upstream | HCON_00049840 | n/a | - |
|  |  |  |  |  |  |  |  |  | Downstream | HCON_00049830 | n/a | - |
|  |  |  |  |  |  |  |  |  | Intergenic | HCON_00049830-HCON_00049840 | | |
| 5 | 25384592 | T | C | T | 0:141:35:0 | 0:158:2:0 | 0:121:108:0 | 0.03201 | Downstream | HCON_00147350 | n/a | - |
|  |  |  |  |  |  |  |  |  | Intron | HCON_00147340 | n/a | - |

Nucleotide position (Pos) in chromosome (Ch); reference (Ref), alternative (Alt), and resistance-associated (Res) alleles; nucleotide base counts (A:T:C:G) in unselected (US) and monepantel-selected (S) *Haemonchus contortus* populations obtained after crossing parental susceptible with parental resistant (Par-R) isolates, using susceptible males and resistant females (SR); adjusted *P*-value (*P*adj) corrected for multiple testing; annotation (Ann) of variants; gene name from WormBase ParaSite (http://parasite.wormbase.org); and orthologous gene in *Caenorhabditis elegans* from WormBase (https://wormbase.org)
