# Supplementary material for: Extreme-QTL mapping of monepantel resistance in Haemonchus contortus
Source: Parasit Vectors. 2019 Aug 14;12:403. doi: 10.1186/s13071-019-3663-9 (PMC6693152; doi:10.1186/s13071-019-3663-9)
Supplement: Supplementary file 2 — Additional file 2: Table S2. Significant variants detected by X-QTL mapping of monepantel resistance in Haemonchus contortus from the RS population. [file 13071_2019_3663_MOESM2_ESM.docx]

**Additional file 2: Table S2** Significant variants detected by X-QTL mapping of monepantel resistance in *Haemonchus contortus* from the RS population

| **Ch** | **Pos** | **Ref** | **Alt** | **Res** | **US-RS** | **S-RS** | **Par-R** | ***P*adj** | **Ann** | **Gene** | **Name** | ***C. elegans***  **orthologous** | |  |
| --- | --- | --- | --- | --- | --- | --- | --- | --- | --- | --- | --- | --- | --- | --- |
| 2 | 4403522 | T | A | T | 64:26:0:0 | 34:74:0:0 | 47:57:0:0 | 0.03410 | Intergenic | HCON_00036620- | n/a | C09F9.2 | |  |
|  |  |  |  |  |  |  |  |  |  | HCON_00036630 | n/a | K02B7.3 | |  |
| 2 | 7013333 | C | T | T | 0:22:88:1 | 0:64:42:0 | 0:83:37:3 | 0.00368 | Synonymous | HCON_00038600 | n/a | T27D12.1 | |  |
| 2 | 7013506 | A | G | G | 79:0:2:21 | 35:0:10:59 | 41:2:3:63 | 0.00691 | Intron |  |  |  | |  |
| 2 | 7080772 | T | C | C | 0:40:12:0 | 0:17:48:0 | 0:16:58:0 | 0.03555 | Intergenic | HCON_00038620- | n/a | - | |  |
|  |  |  |  |  |  |  |  |  |  | HCON_00038630 | n/a | amt-3 | |  |
| 2 | 7263584 | T | C | T | 0:53:47:0 | 0:94:10:0 | 0:88:14:0 | 0.00368 | Upstream | HCON_00038810 | n/a | Y57A10A.13 | |  |
|  |  |  |  |  |  |  |  |  | Intron | HCON_00038800 | n/a | F07F6.8, F07F6.7 | |  |
| 2 | 7268492 | G | A | A | 39:0:0:57 | 73:0:0:19 | 61:0:0:30 | 0.04186 | Downstream | HCON_00038800 | n/a | F07F6.8, F07F6.7 | |  |
|  |  |  |  |  |  |  |  |  | Intron | HCON_00038810 | n/a | Y57A10A.13 | |  |
| 2 | 7268501 | T | G | G | 0:58:0:41 | 0:19:0:77 | 0:29:0:62 | 0.03344 | Splice & Intron | HCON_00038810 | n/a | Y57A10A.13 | |  |
|  |  |  |  |  |  |  |  |  | Downstream | HCON_00038800 | n/a | F07F6.8, F07F6.7 | |  |
| 2 | 7309257 | C | G | G | 0:0:70:14 | 0:0:35:46 | 0:0:29:50 | 0.04186 | Upstream | HCON_00038850 | n/a | cup-15 | |  |
| 2 | 7309258 | G | A | A | 14:0:0:71 | 46:0:0:36 | 51:0:0:27 | 0.04212 | Upstream | HCON_00038860 | n/a | sdhb-1 | |  |
| 2 | 7309342 | A | T | T | 84:19:0:0 | 36:47:0:0 | 39:50:0:0 | 0.04530 | Intergenic | HCON_00038850-HCON_00038860 | | |  |  |
| 2 | 7309352 | T | C | C | 0:80:19:0 | 0:35:49:0 | 0:36:51:0 | 0.03924 |  |  |  |  | |  |
| 2 | 7320214 | C | G | G | 0:0:45:15 | 0:0:17:47 | 0:0:18:72 | 0.04977 | Upstream | HCON_00038880 | Succinate dehydrogenase [ubiquinone] iron-sulfur subunit, mitochondrial | sdhb-1 | |  |
|  |  |  |  |  |  |  |  |  | Downstream | HCON_00038870 | n/a | sdhb-1 | |  |
|  |  |  |  |  |  |  |  |  | Intergenic | HCON_00038870-HCON_00038880 | | |  | |
| 2 | 7350448 | C | T | C | 0:63:28:1 | 0:37:80:0 | 0:28:76:0 | 0.04212 | Intron | HCON_00038890 | n/a | - | |  |
| 2 | 7385310 | C | G | G | 0:0:71:24 | 1:0:35:63 | 0:0:53:51 | 0.04186 | Synonymous | HCON_00038930 | n/a | C31E10.6, C16A11.2, C31E10.5 | |  |
|  |  |  |  |  |  |  |  |  | Downstream | HCON_00038940 | n/a | - | |  |
| 2 | 7385855 | A | T | T | 47:52:0:0 | 15:100:0:0 | 16:98:0:0 | 0.02969 | Downstream | HCON_00038940 | n/a | - | |  |
|  |  |  |  |  |  |  |  |  | Intron | HCON_00038930 | n/a | C31E10.6, C16A11.2, C31E10.5 | |  |
| 2 | 7505065 | A | G | G | 55:0:0:42 | 17:0:0:83 | 25:0:0:69 | 0.01015 | Synonymous | HCON_00039065 | n/a | M176.4 | |  |
| 2 | 7505077 | G | A | A | 43:0:0:51 | 80:0:0:16 | 76:0:0:23 | 0.04186 | Downstream | HCON_00039070 | n/a | lect-2 | |  |
| 2 | 7699925 | C | T | T | 0:15:77:0 | 0:53:45:0 | 0:77:40:0 | 0.04186 | Downstream | HCON_00039310 | n/a | - | |  |
|  |  |  |  |  |  |  |  |  | Intergenic | HCON_00039300-HCON_00039310 | n/a | - | |  |
| 2 | 7706841 | C | A | A | 10:7:63:0 | 30:6:18:0 | 23:12:27:0 | 0.03251 | Upstream | HCON_00039310 | n/a | - | |  |
|  |  |  |  |  |  |  |  |  | Upstream | HCON_00039320 | n/a | - | |  |
|  |  |  |  |  |  |  |  |  | Upstream | HCON_00039330 | n/a | - | |  |
|  |  |  |  |  |  |  |  |  | Intergenic | HCON_00039320-HCON_00039330 | | |  | |
| 2 | 7732215 | C | A | C | 33:0:68:0 | 2:0:89:0 | 18:0:89:0 | 0.01413 | Intron | HCON_00039350 | n/a | - | |  |
| 2 | 7846702 | T | C | C | 0:114:33:0 | 0:56:68:0 | 0:84:55:0 | 0.03560 | Intron | HCON_00039400 | n/a | - | |  |
| 2 | 7871442 | T | C | C | 0:70:37:0 | 0:25:68:0 | 0:40:57:0 | 0.03924 | Upstream | HCON_00039430 | n/a | - | |  |
|  |  |  |  |  |  |  |  |  | Downstream | HCON_00039450 | n/a | - | |  |
|  |  |  |  |  |  |  |  |  | Intron | HCON_00039400 | n/a | - | |  |
| 2 | 7901367 | A | G | A | 45:0:0:67 | 81:0:0:23 | 74:0:0:20 | 0.02413 | Intron | HCON_00039480 | n/a | - | |  |
| 2 | 7901396 | A | C | C | 68:0:44:0 | 21:0:89:0 | 21:0:74:0 | 0.00104 | Intron |  |  |  | |  |
| 2 | 7901404 | T | G | T | 0:49:0:65 | 0:96:0:23 | 0:74:0:21 | 0.00691 | Intron |  |  |  | |  |
| 2 | 7901424 | A | C | A | 44:0:63:0 | 97:0:19:0 | 65:0:20:0 | 0.00033 | Intron |  |  |  | |  |
| 2 | 7901429 | T | A | T | 62:49:0:0 | 22:98:0:0 | 21:64:0:0 | 0.00622 | Intron |  |  |  | |  |
| 2 | 7901455 | C | T | C | 0:69:47:0 | 0:23:96:0 | 0:21:75:0 | 0.00104 | Intron |  |  |  | |  |
| 2 | 7901477 | G | A | G | 61:0:0:43 | 22:0:0:97 | 17:0:0:74 | 0.00201 | Intron |  |  |  | |  |
| 2 | 7901587 | C | G | G | 0:0:56:32 | 0:0:14:76 | 0:0:10:68 | 0.00033 | Intron |  |  |  | |  |
| 2 | 7935749 | A | G | G | 46:0:0:97 | 17:0:0:175 | 29:0:0:114 | 0.04303 | Intron | HCON_00039490 | n/a | - | |  |
| 2 | 7953516 | T | C | C | 0:75:32:0 | 0:26:57:0 | 0:43:47:0 | 0.04977 | Upstream | HCON_00039500 | n/a | - | |  |
|  |  |  |  |  |  |  |  |  | Intron | HCON_00039510 | n/a | - | |  |
| 2 | 7979349 | G | A | A | 34:0:0:59 | 59:0:0:17 | 51:0:0:47 | 0.04375 | Downstream | HCON_00039540 | n/a | - | |  |
|  |  |  |  |  |  |  |  |  | Intergenic | HCON_00039540-HCON_00039550 | n/a | bicd-1 | |  |
| 2 | 8129986 | T | A | A | 23:85:0:0 | 59:40:0:0 | 78:43:0:0 | 0.02391 | Intron | HCON_00039640 | n/a | zyx-1 | |  |
| 2 | 8265309 | T | C | C | 0:113:75:0 | 0:47:147:0 | 0:64:155:0 | 0.00003 | Upstream | HCON_00039750 | n/a | srx-41 | |  |
| 2 | 8265316 | C | A | A | 58:0:129:0 | 113:0:79:0 | 113:0:104:0 | 0.04186 | Downstream | HCON_00039760 | n/a | - | |  |
|  |  |  |  |  |  |  |  |  | Intergenic | HCON_00039750-HCON_00039760 | | |  | |
| 2 | 8295346 | A | T | T | 72:21:0:0 | 33:65:0:0 | 53:54:0:0 | 0.00368 | Upstream | HCON_00039780 | n/a | - | |  |
|  |  |  |  |  |  |  |  |  | Downstream | HCON_00039790 | n/a | K10B2.4 | |  |
|  |  |  |  |  |  |  |  |  | Downstream | HCON_00039800 | n/a | dhs-7, dhs-8, F32A5.8, E04F6.15, DC2.5, K10H10.6 | |  |
|  |  |  |  |  |  |  |  |  | Intergenic | HCON_00039790-HCON_00039800 | | |  | |
| 2 | 8379504 | C | T | C | 0:46:53:0 | 0:12:87:0 | 0:11:84:0 | 0.04212 | Upstream | HCON_00039930 | n/a | mfn-1 | |  |
| 2 | 8379581 | G | A | A | 42:0:0:66 | 75:0:0:24 | 75:0:0:25 | 0.04212 | Downstream | HCON_00039910 | n/a | - | |  |
|  |  |  |  |  |  |  |  |  | Intron | HCON_00039920 | n/a | W02B12.10 | |  |
| 2 | 8594705 | T | A | T | 45:39:0:0 | 14:79:0:0 | 23:66:1:0 | 0.04186 | Downstream | HCON_00040140 | n/a | B0511.12 | |  |
|  |  |  |  |  |  |  |  |  | Downstream | HCON_00040150 | n/a | C17G10.7 | |  |
|  |  |  |  |  |  |  |  |  | Intergenic | HCON_00040140-HCON_00040150 | | |  | |
| 2 | 8723949 | C | T | C | 0:51:57:0 | 0:11:86:0 | 0:20:82:0 | 0.02391 | Upstream | HCON_00040300 | Protein W02B12.13, isoform b | - | |  |
| 2 | 8723954 | A | C | A | 56:0:48:0 | 89:0:10:0 | 84:0:20:0 | 0.01413 | Intergenic | HCON_00040300- |  |  | |  |
| 2 | 8723978 | T | C | C | 0:56:53:0 | 0:13:82:0 | 0:20:87:0 | 0.01413 |  | HCON_00040310 | n/a | nsy-1 | |  |
| 2 | 8726123 | C | T | T | 0:41:78:0 | 0:71:30:0 | 0:63:40:0 | 0.04977 |  |  |  |  | |  |
| 2 | 8862471 | C | T | T | 0:62:30:0 | 0:88:2:0 | 0:88:24:0 | 0.02391 | Intergenic | HCON_00040340- | n/a | - | |  |
| 2 | 8870929 | A | T | T | 52:119:0:0 | 17:178:0:0 | 18:156:0:0 | 0.04977 |  | HCON_00040350 | n/a | - | |  |
| 2 | 8887895 | C | A | A | 34:0:56:0 | 69:0:20:0 | 67:0:39:0 | 0.04212 |  |  |  |  | |  |
| 2 | 9143955 | C | A | C | 52:0:42:0 | 20:0:87:0 | 31:0:69:0 | 0.04186 | Upstream | HCON_00040550 | n/a | - | |  |
| 2 | 9143973 | A | G | A | 35:0:0:52 | 76:0:0:18 | 65:0:0:27 | 0.02423 | Downstream | HCON_00040530 | n/a | - | |  |
| 2 | 9144005 | T | A | A | 27:58:0:8 | 65:24:0:19 | 64:44:0:7 | 0.04186 | Intron | HCON_00040540 | n/a | - | |  |
| 2 | 9144037 | T | C | T | 0:43:55:0 | 0:98:24:0 | 0:88:30:0 | 0.02442 |  |  |  |  | |  |
| 2 | 9185524 | C | G | G | 0:0:53:45 | 0:0:18:85 | 0:0:38:72 | 0.04186 | Intergenic | HCON_00040570- | n/a | gtf-2E2 | |  |
|  |  |  |  |  |  |  |  |  |  | HCON_00040580 | n/a | F33G12.6 | |  |
| 2 | 9403401 | C | T | C | 0:34:47:0 | 0:8:101:0 | 0:7:87:0 | 0.01854 | Upstream | HCON_00040700 | n/a | sfxn-1.4 | |  |
| 2 | 9403437 | G | A | G | 32:0:0:49 | 5:0:0:90 | 9:0:0:82 | 0.02385 |  |  |  |  | |  |
| 2 | 10199955 | G | A | A | 30:0:0:59 | 77:0:0:20 | 71:0:0:33 | 0.00104 | Intron | HCON_00041360 | n/a | unc-61 | |  |
| 2 | 26770651 | C | A | C | 52:0:50:0 | 11:0:71:0 | 24:0:75:0 | 0.04186 | Downstream | HCON_00051350 | n/a | F33A8.4 | |  |
|  |  |  |  |  |  |  |  |  | Intergenic | HCON_00051340-HCON_00051350 | n/a | - | |  |
| 3 | 36273321 | C | G | C | 0:0:5:36 | 0:0:27:10 | 0:0:34:20 | 0.03555 | Intron | HCON_00090270 | n/a | frm-8 | |  |

Nucleotide position (Pos) in chromosome (Ch); reference (Ref), alternative (Alt), and resistance-associated (Res) alleles; nucleotide base counts (A:T:C:G) in unselected (US) and monepantel-selected (S) *Haemonchus contortus* populations obtained after crossing parental susceptible with parental resistant (Par-R) isolates, using resistant males and susceptible females (RS); adjusted *P*-value (*P*adj) corrected for multiple testing; annotation (Ann) of variants; gene name from WormBase ParaSite (http://parasite.wormbase.org); and orthologous gene in *Caenorhabditis elegans* from WormBase (https://wormbase.org)
