# Supplementary material for: Extreme-QTL mapping of monepantel resistance in Haemonchus contortus
Source: Parasit Vectors. 2019 Aug 14;12:403. doi: 10.1186/s13071-019-3663-9 (PMC6693152; doi:10.1186/s13071-019-3663-9)
Supplement: Supplementary file 3 — Additional file 3: Table S3. Significant variants detected by X-QTL mapping of monepantel resistance in Haemonchus contortus from both the SR and RS populations. [file 13071_2019_3663_MOESM3_ESM.docx]

**Additional file 3: Table S3** Significant variants detected by X-QTL mapping of monepantel resistance in *Haemonchus contortus* from both the SR and RS populations

| **Ch** | **Pos** | **Ref** | **Alt** | **Res** | **US** | **S** | **Par-R** | ***P*adj** | **Ann** | **Gene** | **Name** | ***C. elegans***  **orthologous** |
| --- | --- | --- | --- | --- | --- | --- | --- | --- | --- | --- | --- | --- |
| 2 | 3074289 | A | T,C | T | 67:63:32:0 | 28:115:29:0 | 30:53:7:0 | 0.02939 | Upstream | HCON_00035720 | n/a | - |
|  |  |  |  |  |  |  |  |  | Downstream | HCON_00035710 | n/a | glt-5 |
|  |  |  |  |  |  |  |  |  | Intergenic | HCON_00035710-HCON_00035720 | | |
| 2 | 3372713 | A | G | G | 119:0:0:24 | 67:0:0:70 | 55:0:0:23 | 0.01583 | Upstream | HCON_00191530 | n/a | M05D6.9 |
| 2 | 3372716 | A | G | G | 121:0:0:24 | 75:0:0:70 | 57:0:0:23 | 0.02838 | Intron | HCON_00035970 | n/a | T13H5.1 |
| 2 | 3372717 | A | G | G | 121:0:0:24 | 77:0:0:69 | 57:0:0:23 | 0.03534 |  |  |  |  |
| 2 | 5967403 | A | G | G | 115:0:0:39 | 78:0:0:93 | 43:0:0:48 | 0.04967 | Upstream | HCON_00037740 | n/a | - |
| 2 | 5967414 | A | C | C | 131:0:45:0 | 86:0:105:0 | 48:0:51:0 | 0.02587 | Downstream | HCON_00037760 | n/a | ilcr-2 |
| 2 | 5967416 | T | C | T | 0:46:132:0 | 0:105:86:0 | 0:49:47:0 | 0.02839 | Intergenic | HCON_00037740-HCON_00037760 | | |
| 2 | 7023941 | A | G | G | 128:0:0:56 | 65:0:0:99 | 39:0:0:50 | 0.02885 | Downstream | HCON_00038610 | n/a | clh-1 |
|  |  |  |  |  |  |  |  |  | Intron | HCON_00038600 | n/a | T27D12.1 |
| 2 | 7080772 | T | C | C | 0:65:43:0 | 0:38:106:0 | 0:16:58:0 | 0.03714 | Intergenic | HCON_00038620-HCON_00038630 | n/a  n/a | -  amt-3 |
| 2 | 7201718 | A | T,C | A | 80:72:32:3 | 138:34:40:2 | 77:19:15:8 | 0.04823 | Upstream | HCON_00038710 | n/a | ddl-2 |
| 2 | 7201767 | A | G | G | 148:0:0:51 | 101:0:0:116 | 63:0:0:72 | 0.01752 | Downstream | HCON_00038730 | n/a | mrpl-37 |
|  |  |  |  |  |  |  |  |  | Intron | HCON_00038720 | n/a | - |
| 2 | 7263078 | A | G | A | 123:1:0:71 | 223:0:0:36 | 105:0:0:17 | 0.02433 | Upstream | HCON_00038800 | n/a | F07F6.8,  F07F6.7 |
|  |  |  |  |  |  |  |  |  | Intergenic | HCON_00038790-HCON_00038800 | n/a | - |
| 2 | 7263584 | T | C | T | 0:120:67:0 | 0:196:26:0 | 0:88:14:0 | 0.02166 | Upstream | HCON_00038810 | n/a | Y57A10A.13 |
|  |  |  |  |  |  |  |  |  | Intron | HCON_00038800 | n/a | F07F6.8,  F07F6.7 |
| 2 | 7268492 | G | A | A | 89:0:0:86 | 145:0:0:40 | 61:0:0:30 | 0.03545 | Downstream | HCON_00038800 | n/a | F07F6.8,  F07F6.7 |
| 2 | 7268493 | C | T | T | 0:88:87:0 | 0:143:40:0 | 0:60:29:0 | 0.03517 | Intron | HCON_00038810 | n/a | Y57A10A.13 |
| 2 | 7268501 | T | G | G | 0:88:0:96 | 0:40:0:151 | 0:29:0:62 | 0.03300 | Splice & Intron | HCON_00038810 | n/a | Y57A10A.13 |
|  |  |  |  |  |  |  |  |  | Downstream | HCON_00038800 | n/a | F07F6.8,  F07F6.7 |
| 2 | 7297894 | G | A | A | 59:1:0:139 | 101:0:0:76 | 66:0:0:45 | 0.04179 | Downstream | HCON_00038830 | n/a | - |
|  |  |  |  |  |  |  |  |  | Downstream | HCON_00038840 | n/a | - |
|  |  |  |  |  |  |  |  |  | Intergenic | HCON_00038830-HCON_00038840 | | |
| 2 | 7298110 | G | A | A | 89:0:0:120 | 132:0:0:53 | 79:0:0:27 | 0.01880 | Synonymous | HCON_00038840 | n/a | - |
| 2 | 7298155 | G | A | A | 77:0:0:92 | 131:0:0:49 | 71:0:0:21 | 0.04967 | Downstream | HCON_00038830 | n/a | - |
| 2 | 7298158 | A | C | C | 97:11:60:0 | 52:9:118:0 | 29:4:60:0 | 0.02442 |  |  |  |  |
| 2 | 7298176 | T | C | C | 0:95:72:0 | 0:50:136:0 | 0:28:66:0 | 0.01880 | Downstream | HCON_00038830 | n/a | - |
| 2 | 7298227 | A | G | G | 98:0:0:42 | 64:0:0:99 | 29:0:0:41 | 0.03661 | Intron | HCON_00038840 | n/a | - |
| 2 | 7298231 | T | G | G | 0:91:0:45 | 0:56:0:100 | 0:21:0:49 | 0.03534 |  |  |  |  |
| 2 | 7300762 | C | A | A | 55:0:89:0 | 111:1:40:0 | 63:0:30:0 | 0.01410 | Upstream | HCON_00038840 | n/a | - |
| 2 | 7300775 | G | A | A | 55:0:0:77 | 113:0:0:35 | 68:0:0:25 | 0.01583 | Downstream | HCON_00038850 | n/a | cup-15 |
|  |  |  |  |  |  |  |  |  | Intergenic | HCON_00038840-HCON_00038850 | | |
| 2 | 7309342 | A | T | T | 124:47:0:0 | 65:93:0:0 | 39:50:0:0 | 0.02442 | Upstream | HCON_00038850 | n/a | cup-15 |
| 2 | 7309343 | C | T | T | 0:51:119:0:0 | 0:95:62:0 | 0:52:37:0 | 0.03534 | Upstream | HCON_00038860 | n/a | sdhb-1 |
| 2 | 7309344 | T | A | A | 47:122:0:4 | 92:61:0:2 | 49:37:0:3 | 0.01904 | Intergenic | HCON_00038850-HCON_00038860 | | |
| 2 | 7309352 | T | C | C | 0:119:47:0 | 0:65:97:0 | 0:36:51:0 | 0.02442 |  |  |  |  |
| 2 | 7309353 | G | A | A | 46:0:0:114 | 97:0:0:65 | 51:0:0:36 | 0.02939 |  |  |  |  |
| 2 | 7309358 | A | C | C | 110:0:51:1 | 62:0:101:1 | 30:0:57:0 | 0.03900 |  |  |  |  |
| 2 | 7309385 | T | C | C | 0:115:60:0 | 0:66:117:0 | 0:32:68:0 | 0.04967 |  |  |  |  |
| 2 | 7320214 | C | G | G | 0:0:76:59 | 0:0:33:116 | 0:0:18:72 | 0.01410 | Upstream | HCON_00038880 | Succinate dehydrogenase [ubiquinone] iron-sulfur subunit, mitochondrial | sdhb-1 |
| 2 | 7320221 | T | A | A | 56:72:0:0 | 114:36:0:0 | 73:19:1:0 | 0.02587 | Downstream | HCON_00038870 | n/a | sdhb-1 |
| 2 | 7320242 | T | C | C | 0:71:56:0 | 0:37:117:0 | 0:16:72:0 | 0.03115 | Intergenic | HCON_00038870-HCON_00038880 | | |
| 2 | 7350138 | G | C | C | 0:0:77:137 | 0:0:128:77 | 0:0:68:41 | 0.02442 | Intron | HCON_00038890 | n/a | - |
| 2 | 7350162 | G | A | A | 73:0:0:135 | 124:0:0:80 | 61:0:0:40 | 0.03534 |  |  |  |  |
| 2 | 7350174 | T | C | C | 0:118:69:15 | 0:71:124:6 | 0:27:64:7 | 0.03227 |  |  |  |  |
| 2 | 7350256 | T | C | T | 0:96:115:10 | 0:141:55:3 | 0:65:24:0 | 0.02442 |  |  |  |  |
| 2 | 7350305 | G | A | G | 102:0:0:102 | 49:0:0:148 | 21:0:0:73 | 0.04179 |  |  |  |  |
| 2 | 7350448 | C | T | T | 0:104:85:1 | 0:67:156:0 | 0:28:76:0 | 0.04393 |  |  |  |  |
| 2 | 7375072 | T | A | A | 114:73:0:0 | 184:34:0:0 | 96:15:0:0 | 0.04712 | Synonymous | HCON_00038920 | n/a | flad-1 |
| 2 | 7375853 | G | A | A | 144:0:0:42 | 178:0:0:7 | 80:0:0:6 | 0.03940 | Upstream | HCON_00038930 | n/a | C31E10.6, C16A11.2, C31E10.5 |
| 2 | 7438328 | T | A | A | 59:147:0:0 | 107:82:0:0 | 49:46:0:0 | 0.02442 | Downstream | HCON_00038980 | n/a | ZK1321.1 |
| 2 | 7438391 | T | C | C | 0:150:43:0 | 0:87:84:0 | 0:38:39:0 | 0.04403 | Intron | HCON_00038990 | n/a | - |
| 2 | 7601517 | T | G | G | 0:62:0:101 | 0:19:0:139 | 0:15:0:70 | 0.03546 | Upstream | HCON_00039170 | n/a | icd-1 |
|  |  |  |  |  |  |  |  |  | Upstream | HCON_00039180 | n/a | C56C10.7 |
|  |  |  |  |  |  |  |  |  | Downstream | HCON_00039155 | n/a | C56C10.9 |
|  |  |  |  |  |  |  |  |  | Intron | HCON_00039160 | n/a | vps-32.1 |
| 2 | 7647978 | T | C | C | 0:147:74:5 | 0:75:112:10 | 0:43:54:2 | 0.03517 | Downstream | HCON_00039220 | n/a | - |
|  |  |  |  |  |  |  |  |  | Downstream | HCON_00039230 | n/a | - |
|  |  |  |  |  |  |  |  |  | Intergenic | HCON_00039220-HCON_00039230 | | |
| 2 | 7706841 | C | A | A | 23:14:97:0 | 62:15:51:0 | 23:12:27:0 | 0.02925 | Upstream | HCON_00039310 | n/a | - |
|  |  |  |  |  |  |  |  |  | Upstream | HCON_00039320 | n/a | - |
|  |  |  |  |  |  |  |  |  | Upstream | HCON_00039330 | n/a | - |
|  |  |  |  |  |  |  |  |  | Intergenic | HCON_00039320-HCON_00039330 | | |
| 2 | 7756689 | C | G | C | 0:0:79:102 | 0:0:134:52 | 0:0:47:31 | 0.02587 | Upstream | HCON_00039360 | Acetylcholine receptor monepantel-1 | acr-20 |
|  |  |  |  |  |  |  |  |  | Intergenic | HCON_00039360-HCON_00039370 | Acetylcholine receptor DEG-3-like protein | deg-3 |
| 2 | 7848125 | A | T | T | 130:90:0:0 | 74:153:0:0 | 62:66:0:0 | 0.02360 | Intron | HCON_00039400 | n/a | - |
| 2 | 7848293 | C | T | T | 0:100:141:0 | 0:176:87:0 | 0:75:56:0 | 0.01880 |  |  |  |  |
| 2 | 7858806 | T | C | C | 0:101:83:0 | 0:55:148:0 | 0:27:69:0 | 0.02939 | Upstream | HCON_00039410 | n/a | - |
| 2 | 7859227 | T | A | A | 77:103:0:0 | 129:49:0:0 | 74:27:0:0 | 0.01880 | Intron | HCON_00039400 | n/a | - |
| 2 | 7859228 | T | G | G | 0:103:6:71 | 0:52:8:121 | 0:27:8:64 | 0.02939 |  |  |  |  |
| 2 | 7859502 | G | C | C | 0:0:97:99 | 0:0:148:47 | 0:0:67:25 | 0.03424 |  |  |  |  |
| 2 | 7860926 | A | G | G | 98:0:0:92 | 53:0:0:155 | 31:0:1:74 | 0.03661 | Intron | HCON_00039400 | n/a | - |
| 2 | 7879208 | A | C | C | 101:0:76:0 | 52:0:128:0 | 35:0:48:0 | 0.03571 |  |  |  |  |
| 2 | 7888631 | T | C | C | 0:123:98:0 | 0:64:152:0 | 0:51:69:0 | 0.02939 | Downstream | HCON_00039470 | n/a | - |
|  |  |  |  |  |  |  |  |  | Intron | HCON_00039400 | n/a | - |
| 2 | 7901336 | C | T | C | 0:96:103:0 | 0:42:151:0 | 1:22:77:0 | 0.03115 | Intron | HCON_00039480 | n/a | - |
| 2 | 7901337 | A | G | A | 98:0:0:94 | 151:1:0:42 | 78:0:0:20 | 0.02592 |  |  |  |  |
| 2 | 7901367 | A | G | A | 94:0:0:104 | 143:0:0:45 | 74:0:0:20 | 0.01880 |  |  |  |  |
| 2 | 7901396 | A | C | C | 110:0:97:0 | 46:0:150:0 | 21:0:74:0 | 0.00693 |  |  |  |  |
| 2 | 7901404 | T | G | T | 0:101:0:106 | 0:159:0:48 | 0:74:0:21 | 0.01583 |  |  |  |  |
| 2 | 7901424 | A | C | A | 101:0:102:0 | 162:0:42:0 | 65:0:20:0 | 0.00505 |  |  |  |  |
| 2 | 7901429 | T | A | T | 101:108:0:0 | 46:168:0:0 | 21:64:0:0 | 0.01625 |  |  |  |  |
| 2 | 7901455 | C | T | C | 0:111:101:0 | 0:49:165:0 | 0:21:75:0 | 0.00505 |  |  |  |  |
| 2 | 7901477 | G | A | G | 99:0:0:95 | 49:0:0:166 | 17:0:0:74 | 0.01410 |  |  |  |  |
| 2 | 7901587 | C | G | G | 0:0:92:71 | 0:0:34:135 | 0:0:10:68 | 0.00053 |  |  |  |  |
| 2 | 7901703 | A | G | A | 74:0:0:101 | 116:0:0:44 | 60:0:0:25 | 0.02587 |  |  |  |  |
| 2 | 7901788 | C | T | T | 0:51:90:0 | 0:92:40:0 | 0:35:22:0 | 0.03115 |  |  |  |  |
| 2 | 7902759 | A | G | G | 80:0:0:31 | 33:0:0:69 | 14:0:0:28 | 0.01880 |  |  |  |  |
| 2 | 7902809 | T | C | C | 0:79:48:0 | 0:35:88:0 | 0:21:36:0 | 0.04823 |  |  |  |  |
| 2 | 7902820 | T | A | A | 46:80:0:0 | 91:40:0:0 | 33:21:0:0 | 0.04967 |  |  |  |  |
| 2 | 7942190 | C | T | T | 0:159:108:0 | 0:226:55:0 | 0:104:18:0 | 0.02838 | Synonymous | HCON_00039490 | n/a | - |
| 2 | 7954381 | A | G | G | 114:0:0:87 | 49:0:0:121 | 42:0:0:70 | 0.03546 | Upstream | HCON_00039500 | n/a | - |
|  |  |  |  |  |  |  |  |  | Intron | HCON_00039510 | n/a | - |
| 2 | 7958674 | C | T | T | 0:137:148:2 | 0:227:92:0 | 0:98:57:1 | 0.01583 | Intron | HCON_00039510 | n/a | - |
| 2 | 7965637 | T | C | C | 0:116:79:0 | 0:69:139:0 | 0:40:48:0 | 0.03290 | Downstream | HCON_00039510 | n/a | - |
|  |  |  |  |  |  |  |  |  | Downstream | HCON_00039520 | n/a | - |
|  |  |  |  |  |  |  |  |  | Downstream | HCON_00039530 | n/a | - |
|  |  |  |  |  |  |  |  |  | Intergenic | HCON_00039510-HCON_00039520 | | |
| 2 | 7968762 | T | C | C | 0:125:77:0 | 0:74:135:0 | 0:25:71:0 | 0.03714 | Upstream | HCON_00039540 | n/a | - |
|  |  |  |  |  |  |  |  |  | Downstream | HCON_00039530 | n/a | - |
|  |  |  |  |  |  |  |  |  | Intron | HCON_00039520 | n/a | - |
| 2 | 7979797 | G | A | A | 94:0:0:103 | 152:0:0:43 | 61:0:0:28 | 0.00589 | Intergenic | HCON_00039540-HCON_00039550 | n/a | - |
| 2 | 7980020 | A | G | G | 93:0:0:94 | 40:0:0:162 | 31:0:0:52 | 0.00589 |  |  | n/a | bicd-1 |
| 2 | 8015448 | C | T | C | 0:41:119:0 | 0:14:206:0 | 0:7:135:0 | 0.03782 | Upstream | HCON_00039570 | n/a | ZK1248.19 |
|  |  |  |  |  |  |  |  |  | Upstream | HCON_00039580 | n/a | - |
|  |  |  |  |  |  |  |  |  | Intron | HCON_00039560 | n/a | Y53F4B.13 |
| 2 | 8020062 | T | C | C | 0:134:51:0 | 0:67:86:0 | 0:45:46:0 | 0.03546 | Upstream | HCON_00039560 | n/a | Y53F4B.13 |
|  |  |  |  |  |  |  |  |  | Upstream | HCON_00039580 | n/a | - |
| 2 | 8020068 | T | G | G | 0:131:0:52 | 0:67:0:88 | 0:44:0:47 | 0.03900 | Downstream | HCON_00039570 | n/a | ZK1248.19 |
|  |  |  |  |  |  |  |  |  | Intergenic | HCON_00039570-HCON_00039580 | | |
| 2 | 8032604 | C | T | T | 0:38:71:23 | 0:73:29:21 | 0:31:19:13 | 0.04823 | Upstream | HCON_00039590 | n/a | - |
|  |  |  |  |  |  |  |  |  | Upstream | HCON_00192080 | n/a | - |
|  |  |  |  |  |  |  |  |  | Upstream | HCON_00039600 | n/a | - |
|  |  |  |  |  |  |  |  |  | Downstream | HCON_00039580 | n/a | - |
|  |  |  |  |  |  |  |  |  | Intergenic | HCON_00039590-HCON_00192080 | | |
| 2 | 8039609 | G | A | A | 58:0:0:95 | 117:0:0:56 | 51:0:0:23 | 0.03661 | Upstream | HCON_00039610 | n/a | - |
|  |  |  |  |  |  |  |  |  | Downstream | HCON_00039600 | n/a | - |
|  |  |  |  |  |  |  |  |  | Intergenic | HCON_00039600-HCON_00039610 | | |
| 2 | 8044391 | C | T | T | 0:51:121:0 | 0:113:86:0 | 0:39:45:0 | 0.04931 | Synonymous | HCON_00039610 | n/a | - |
| 2 | 8044400 | T | C | C | 0:123:55:0 | 0:80:113:0 | 0:44:38:0 | 0.03534 |  |  |  |  |
| 2 | 8129986 | T | A | A | 64:152:0:0 | 116:93:0:0 | 78:43:0:0 | 0.03546 | Intron | HCON_00039640 | n/a | zyx-1 |
| 2 | 8140991 | T | A | A | 88:75:0:0 | 142:35:0:0 | 71:25:0:1 | 0.04823 |  |  |  |  |
| 2 | 8164572 | T | C | C | 0:89:109:0 | 0:42:166:0 | 0:17:79:0 | 0.04314 | Synonymous | HCON_00039660 | n/a | F18A11.3 |
|  |  |  |  |  |  |  |  |  | Downstream | HCON_00039670 | n/a | F18A11.2 |
| 2 | 8167428 | G | A | A | 67:0:0:46 | 99:0:0:10 | 55:0:0:1 | 0.03714 | Downstream | HCON_00039660 | n/a | F18A11.3 |
|  |  |  |  |  |  |  |  |  | Intron | HCON_00039670 | n/a | F18A11.2 |
| 2 | 8180204 | A | G | G | 46:0:0:50 | 9:0:0:74 | 4:0:0:57 | 0.03900 | Downstream | HCON_00039680 | n/a | - |
|  |  |  |  |  |  |  |  |  | Intergenic | HCON_00039670-HCON_00039680 | n/a | F18A11.2 |
| 2 | 8379504 | C | T | C | 0:60:126:0 | 0:20:186:0 | 0:11:84:0 | 0.03517 | Upstream | HCON_00039930 | n/a | mfn-1 |
|  |  |  |  |  |  |  |  |  | Downstream | HCON_00039910 | n/a | - |
|  |  |  |  |  |  |  |  |  | Intron | HCON_00039920 | n/a | W02B12.10 |
| 2 | 8388929 | G | C | C | 3:0:55:81 | 1:0:121:49 | 0:0:65:35 | 0.03661 | Downstream | HCON_00039950 | n/a | rga-1 |
| 2 | 8389219 | T | A | A | 79:104:0:0 | 143:54:0:0 | 77:34:0:0 | 0.01583 | Intron | HCON_00039940 | Iron sulphur-containing domain containing protein | cisd-1 |
| 2 | 8436025 | G | A | A | 70:0:0:91 | 141:0:0:58 | 43:0:0:30 | 0.04931 | Intron | HCON_00039980 | n/a | cyd-1 |
| 2 | 8436027 | G | A | A | 73:0:0:94 | 143:0:0:57 | 45:0:0:31 | 0.03534 |  |  |  |  |
| 2 | 8483918 | A | T | A | 109:66:0:0 | 162:22:0:0 | 87:13:0:0 | 0.01880 | Upstream | HCON_00040060 | n/a | - |
|  |  |  |  |  |  |  |  |  | Downstream | HCON_00040050 | n/a | - |
|  |  |  |  |  |  |  |  |  | Intergenic | HCON_00040040-HCON_00040050 | n/a | C44B7.7 |
| 2 | 8542891 | T | A | A | 55:123:0:0 | 122:86:0:0 | 54:50:0:0 | 0.02587 | Upstream | HCON_00040090 | Basic helix-loop-helix dimerisation region bHLH domain containing protein | - |
|  |  |  |  |  |  |  |  |  | Downstream | HCON_00040100 | n/a | - |
|  |  |  |  |  |  |  |  |  | Intergenic | HCON_00040100-HCON_00040110 | n/a | - |
| 2 | 8603610 | T | G | G | 0:37:0:136 | 0:7:0:199 | 0:2:0:93 | 0.03365 | Downstream | HCON_00040160 | n/a | - |
|  |  |  |  |  |  |  |  |  | Intron | HCON_00040150 | n/a | C17G10.7 |
| 2 | 8605260 | T | C | C | 0:104:104:0 | 0:53:169:0 | 0:31:85:0 | 0.01880 | Intron | HCON_00040150 | n/a | C17G10.7 |
|  |  |  |  |  |  |  |  |  | Intron | HCON_00040160 | n/a | - |
| 2 | 8668902 | G | A | A | 59:0:0:95 | 115:0:0:55 | 41:0:0:31 | 0.04823 | Downstream | HCON_00040220 | n/a | spi-1 |
|  |  |  |  |  |  |  |  |  | Intergenic | HCON_00040220-HCON_00040230 | n/a | - |
| 2 | 8695492 | T | C | C | 0:87:73:0 | 0:50:139:0 | 0:33:60:0 | 0.03607 | Upstream | HCON_00040260 | n/a | - |
|  |  |  |  |  |  |  |  |  | Intron | HCON_00040250 | n/a | - |
| 2 | 8723954 | A | C | A | 97:0:91:0 | 158:0:50:0 | 84:0:20:0 | 0.03661 | Upstream | HCON_00040300 | Protein W02B12.13, isoform b | - |
| 2 | 8726118 | T | C | T | 0:140:81:0 | 0:180:28:0 | 0:91:10:0 | 0.02925 | Intergenic | HCON_00040300-HCON_00040310 | n/a | nsy-1 |
| 2 | 8726177 | C | T | C | 0:72:139:0 | 0:24:183:0 | 0:12:94:0 | 0.03115 |  |  |  |  |
| 2 | 8727463 | C | T | C | 0:151:53:0 | 0:97:102:0 | 0:49:68:0 | 0.03300 |  |  |  |  |
| 2 | 8858415 | C | T | T | 0:127:63:0 | 0:241:33:0 | 0:90:17:0 | 0.03517 | Intergenic | HCON_00040340-HCON_00040350 | n/a | - |
| 2 | 8858461 | A | G | G | 64:0:0:151 | 29:1:0:255 | 14:0:0:119 | 0.03534 |  |  | n/a | - |
| 2 | 8858464 | T | A | A | 158:61:0:5 | 264:26:0:2 | 114:14:0:5 | 0.03115 |  |  |  |  |
| 2 | 8881493 | C | A | A | 67:0:108:2 | 127:0:63:3 | 64:0:28:6 | 0.02995 |  |  |  |  |
| 2 | 9386129 | G | A | A | 93:0:0:68 | 158:0:0:28 | 76:0:0:15 | 0.02151 | Upstream | HCON_00040690 | n/a | - |
| 2 | 9387118 | G | A | G | 52:0:0:126 | 12:0:1:178 | 2:0:0:108 | 0.01880 | Intron |  |  |  |
| 2 | 9387154 | G | A | A | 95:0:0:74 | 154:0:0:28 | 85:0:0:10 | 0.01583 |  |  |  |  |
| 2 | 9387250 | G | T | T | 0:101:0:75 | 0:156:0:33 | 0:85:0:5 | 0.04823 |  |  |  |  |
| 2 | 9388260 | A | G | G | 97:0:0:106 | 47:0:0:161 | 31:0:0:72 | 0.03290 | Intron | HCON_00040690 | n/a | - |
| 2 | 9403401 | C | T | C | 0:36:124:1 | 0:9:177:0 | 0:7:87:0 | 0.02838 | Upstream | HCON_00040700 | n/a | sfxn-1.4 |
| 2 | 9403437 | G | A | G | 34:0:0:135 | 6:0:0:176 | 9:0:0:82 | 0.03545 | Intergenic | HCON_00040700-HCON_00040710 | n/a | K07E1.1 |
| 2 | 9549215 | C | G | G | 0:0:127:77 | 0:0:78:139 | 0:0:29:70 | 0.01904 | Intergenic | HCON_00040860-HCON_00040870 | n/a | - |
| 2 | 9549379 | T | C | C | 12:114:85:0 | 10:61:138:0 | 0:26:64:0 | 0.02360 |  |  | n/a | - |
| 2 | 9885890 | C | G,T | C | 0:5:48:67 | 0:9:82:30 | 0:2:46:4 | 0.03714 | Intergenic | HCON_00041080-HCON_00041090 | n/a | - |
| 2 | 9885892 | G | A | G | 68:0:0:57 | 30:0:0:97 | 4:0:0:49 | 0.04179 |  |  | n/a | - |
| 3 | 1647363 | G | A | A | 9:0:0:75 | 47:0:0:50 | 42:0:0:0 | 0.03914 | Missense | HCON_00077020 | n/a | - |
|  |  |  |  |  |  |  |  |  | Upstream | HCON_00077030 | n/a | cey-2, cey-3 |
| 4 | 2863865 | G | T | G | 0:72:0:103 | 0:26:0:151 | 97:0:0:8 | 0.03009 | Intron | HCON_00116460 | n/a | unc-82 |

Nucleotide position (Pos) in chromosome (Ch); reference (Ref), alternative (Alt), and resistance-associated (Res) alleles; nucleotide base counts (A:T:C:G) in unselected (US) and monepantel-selected (S) *Haemonchus contortus* populations obtained after reciprocal crossing of parental susceptible and resistant (Par-R) males and females, resulting in SR and RS progenies; adjusted *P*-value (*P*adj) corrected for multiple testing; annotation (Ann) of variants; gene name from WormBase ParaSite (http://parasite.wormbase.org); and orthologous gene in *Caenorhabditis elegans* from WormBase (https://wormbase.org)
