# Supplementary material for: Extreme-QTL mapping of monepantel resistance in Haemonchus contortus
Source: Parasit Vectors. 2019 Aug 14;12:403. doi: 10.1186/s13071-019-3663-9 (PMC6693152; doi:10.1186/s13071-019-3663-9)
Supplement: Supplementary file 4 — Additional file 4: Table S4. Sample sequencing data available at the European Nucleotide Archive repository (study accession PRJEB33301). [file 13071_2019_3663_MOESM4_ESM.docx]

**Additional file 4: Table S4** Sample sequencing data available at the European Nucleotide Archive repository (study accession PRJEB33301)

| **Sample name** | **Sample sequence name submitted to the ENA** | | **Sample accession** | **Secondary sample accession** | **Experiment accession** | **Run accession** |
| --- | --- | --- | --- | --- | --- | --- |
|  | **Forward** | **Reverse** |  |  |  |  |
| Par-R | Par326R_HJ3FYCCXY_L6_1.fq.gz | Par326R_HJ3FYCCXY_L6_2.fq.gz | ERS3559771 | SAMEA5756317 | ERX3439321 | ERR3415793 |
| US-SR | Cont412_HKCLKCCXY_L8_1.fq.gz | Cont412_HKCLKCCXY_L8_2.fq.gz | ERS3559772 | SAMEA5756318 | ERX3439322 | ERR3415794 |
| US-RS | Cont383_HKCLKCCXY_L8_1.fq.gz | Cont383_HKCLKCCXY_L8_2.fq.gz | ERS3559773 | SAMEA5756319 | ERX3439323 | ERR3415795 |
| S-SR | Trat412_HJ3FYCCXY_L7_1.fq.gz | Trat412_HJ3FYCCXY_L7_2.fq.gz | ERS3559774 | SAMEA5756320 | ERX3439324 | ERR3415796 |
| S-RS | Trat383_HKCLKCCXY_L8_1.fq.gz | Trat383_HKCLKCCXY_L8_2.fq.gz | ERS3559775 | SAMEA5756321 | ERX3439325 | ERR3415797 |

Unselected (US) and monepantel-selected (S) *Haemonchus contortus* populations obtained in the F3 generation after reciprocal crossing of parental susceptible and parental resistant (Par-R) isolates, crossing susceptible males with resistant females (SR) and resistant males with susceptible females (RS)
